# The Infectivity of Tabardillo or Mexican Typhus

**Published:** 1910-03

**Authors:** 


### The Infectivity of Tabardillo or Mexican Typhus

FOR some time a disease thought to be typhus fever and known among the Mexican physicians as tabardillo, has been prevalent on the plateaus of certain parts of Mexico. There has been some question as to whether this disease was identical with the Rocky Mountain spotted fever of Montana and Idaho.

The article appearing this week is the third one on this subject published in the Public Health Reports.

The first note (December 10, 1909,) reports the finding by inoculation into monkeys and guinea pigs that the typhus fever of Mexico is an entirely distinct disease from Rocky Mountain spotted fever. In addition to the differences found by animal experimentation, these two diseases differ clinically.

The second note (December 24, 1909,) reports the experimental production of fever by the inoculation of blood from cases of human tabardillo in two species of monkeys, the *Macacus rhesus* and the *Cebus capuchinus*. It was also found that cultures made with blood from human cases of the disease gave negative results in every instance on ordinary media.

In the present paper, the authors report the results of the testing of the immunity of the animals reported in the first note. When tested for immunity three or four weeks after the attack of experimental fever, these animals were immune to an injection of virulent blood from cases of human tabardillo; whereas, the inoculation of 6 c. c. of similar blood in a previously untreated monkey was sufficient to produce a typical febrile reaction after an incubation period of eight days.

Filtration experiments showed that the cause of the disease would not pass through a Berkefeld filter and that virulent blood when passed through such a filter ceased to produce the disease in monkeys. The authors believe that the disease is not ordinarily contagious but that there is strong evidence that the disease is spread from man to man by the body louse.

---

Most physicians nowadays regard bowling as an innocent and healthful exercise when taken or indulged in with discretion or within proper limitations. It is, however, generally impossible for others than clubmen to obtain full benefit from the exercise on good bowling alleys located in appropriate surroundings. Now, bowling alleys have been established at 39-41 Goodell Street, Buffalo, under the management of Mr. C. E. Ament, free from all objections. The alleys are high class, no liquor is sold on the premises, and women may visit the place and participate in the game without coming in contact with anything or any person that would jar a refined woman's sense of propriety. Physicians may recommend such of their patients as need exercise of this kind to Mr. Ament's bowling alleys, with a feeling of confidence that they will be well pleased.

---

An office building for physicians is being constructed at the corner of Delaware Avenue and Allen street, Buffalo, to be completed by May 1, 1910. Mr. E. S. Carroll, the owner, will arrange offices to suit tenants if leases are secured long enough before completion of the structure to enable him to make the necessary partitions.

The Cobb Building, in Seattle, is designed for similar purposes, only physicians and dentists being admitted as tenants. It is one of the most elaborate and completely outfitted buildings for professional tenants yet constructed. Amongst its special equipment in every office is compressed air, a vacuum cleaning system, walls with rounded corners, ventilating strips to provide for ventilation without a draft, and many minor details not necessary to mention. The building will be provided with Turkish and hydrotherapeutic baths, lunch room, barber shop, reading room, and supply house for physicians and dentists. None but an ethical practitioner is permitted to rent space.

---

The following rules relating to the cultivation and shipment of oysters, have been promulgated by the government under the pure food and drugs law:

The pollution of oysters with brackish water for fattening purposes is easily detected by bacteriological examination, and when found in that condition hereafter they will be condemned and destroyed.

The receptacles in which oysters and other shellfish are shipped must be cleansed and sterilised as soon as emptied. It is also ruled to be unlawful to ship or sell in interstate commerce oysters to which water has been added, either directly or in the form of ice.

In view of the fact that the shipping season has begun and shippers will need several months to provide themselves with suitable containers for the shipment of shellfish out of contact with ice, no prosecutions will be recommended prior to May 1, 1910.

---

Danger of food infection from the thermos bottle is pointed out the vacuum flask which has recently come into general use to keep hot fluids hot and cold ones cold, makes a very good incubator for bacteria. The danger of keeping contaminated foods warm in such a container is readily seen. Dr. H. E. Tuley, of Louisville, recently experimented with one of these bottles after a baby under his care had been made sick from its use (*Arch. Pediatrics,* August, 1909). He put a feeding in the bottle, and left one in its usual place over night. In the morning a bacteriological count was made. The feeding which had been kept cool contained 3,500 per cu.mm; that in the vacuum flask contained 1,400,000. It can hardly be doubted that there is decided danger in many foods if kept in this way. If put in while sterile, for example, just after being boiled, the danger could be averted; while things kept hot should undoubtedly suffer no harm.—*Detroit Medical Journal.*

---

Chicago is to rejoice in quieter times. The city council has passed the anti-noise ordinance, prohibiting peddlers and hucksters from shouting their wares on the streets.

